# Genomic studies on *Strongyloides stercoralis* in northern and western Thailand

**DOI:** 10.1186/s13071-020-04115-0

**Published:** 2020-05-13

**Authors:** Kittipat Aupalee, Adulsak Wijit, Kittikhun Singphai, Christian Rödelsperger, Siyu Zhou, Atiporn Saeung, Adrian Streit

**Affiliations:** 1 Graduate Doctoral Degree Program in Parasitology, Faculty of Medicine, Chiang Mai University, Chiang Mai, Thailand; 2 Office of Disease Prevention and Control, 1st Department of Disease Control, Ministry of Public Health, Chiang Mai, Thailand; 3 Department of Parasitology, Faculty of Medicine, Chiang Mai University, Chiang Mai, Thailand; 4 Department of Integrative Evolutionary Biology, Max Planck Institute for Developmental Biology, Tübingen, Baden-Württemberg Germany; 5 School of Preclinical Medicine, Guangxi Medical University, Nanning, Guangxi China

**Keywords:** Strongyloidiasis, *Strongyloides stercoralis*, Neglected tropical disease, *SSU*, *cox*1, Phylogeny

## Abstract

**Background:**

Strongyloidiasis is a soil borne helminthiasis, which in most cases is caused by *Strongyloides stercoralis.* Human infections with *S. fuelleborni fuelleborni* and *S. fuelleborni kellyi* also occur. Although up to 370 million people are currently estimated to be infected with *S. stercoralis*, this parasite is frequently overlooked. *Strongyloides stercoralis* is prevalent among humans in Thailand; however, *S. fuelleborni fuelleborni* has also been reported. Three recent genomic studies of individual *S. stercoralis* worms found genetically diverse populations of *S. stercoralis*, with comparably low heterozygosity in Cambodia and Myanmar, and less diverse populations with high heterozygosity in Japan and southern China that presumably reproduce asexually.

**Methods:**

We isolated individual *Strongyloides* spp. from different localities in northern and western Thailand and determined their nuclear small ribosomal subunit rDNA (*18S* rDNA, *SSU*), in particular the hypervariable regions I and IV (HVR-I and HVR-IV), mitochondrial cytochrome *c* oxidase subunit 1 (*cox*1) and for a subset whole genome sequences. These sequences were then compared with each other and with published sequences from different geographical locations.

**Results:**

All 237 worms isolated from 16 different human hosts were *S. stercoralis*, no *S. fuelleborni* was found. All worms had the common *S. stercoralis SSU* HVR IV haplotype A. Two different *SSU* HVR I haplotypes (I and II), both previously described in *S. stercoralis*, were found. No animal heterozygous for the two haplotypes was identified. Among the twelve *cox*1 haplotypes found, five had not been previously described. Based upon the mitochondrial *cox*1 and the nuclear whole genome sequences, *S. stercoralis* in Thailand was phylogenetically intermixed with the samples from other Southeast Asian countries and did not form its own branch. The genomic heterozygosity was even slightly lower than in the samples from the neighboring countries.

**Conclusions:**

In our sample from humans, all *Strongyloides* spp. were *S. stercoralis*. The *S. stercoralis* from northern and western Thailand appear to be part of a diverse, intermixing continental Southeast Asian population. No obvious indication for genetic sub-structuring of *S. stercoralis* within Thailand or within the Southeast Asian peninsula was detected.
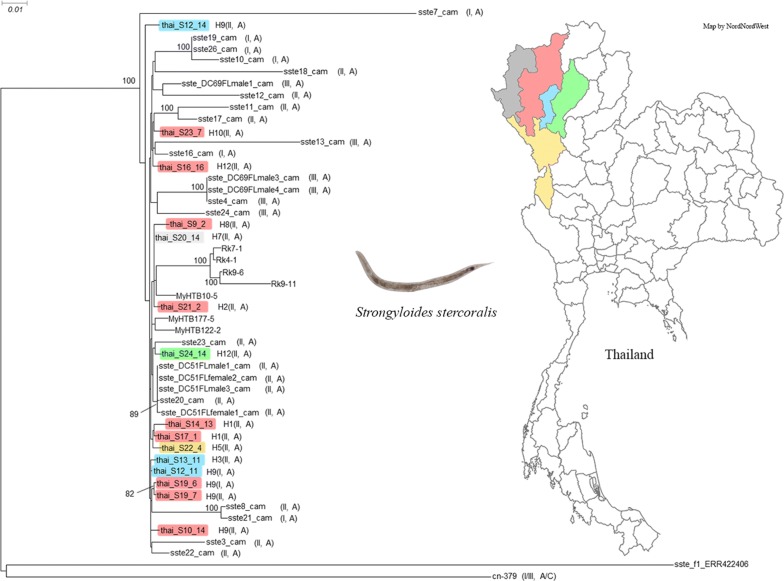

## Background

Strongyloidiasis is a soil-transmitted helminthiasis (STH) [1]. Although it is recognized as one of the neglected tropical diseases (NTDs), it is often overlooked in comparison with other STHs and has therefore by some authors been considered as one of the most neglected NTDs [2–4]. Current estimates of the number of people currently infected with *Strongyloides stercoralis*, the species which causes the vast majority of human strongyloidiasis cases, range from “30–100 million” [1, 5] to “at least 370 million” [3]. Given the difficulties with diagnosis, [1, 6–8] the true number may even be considerably higher. Human infections with two other species of *Strongyloides*, i.e. *S. fuelleborni fuelleborni* and *S. fuelleborni kellyi*, have also been reported [1]. Although described as two subspecies of the species *S. fuelleborni* [9], molecular phylogeny suggests that they are in fact different species [10]. While *S. fuelleborni fuelleborni* has been found on multiple continents and human infections are generally considered to be zoonotic from non-human primates, *S. fuelleborni kellyi* appears to be restricted to Papua New Guinea, and so far no animal host has been identified [1].

Over the past few years, the nuclear *SSU* HVR I, HVR IV and the mitochondrial *cox*1 loci have emerged as the standard markers for molecular taxonomy within the genus *Strongyloides* and within the species *S. stercoralis* [11–21], and a nomenclature system for the different haplotypes has been proposed and extended. Very recently Barratt et al. [22] compiled the different haplotypes reported by various authors and presented a global survey of *S. stercoralis* and *S. fuelleborni* genotypes including newly determined and previously published sequences. A few studies, all analyzing samples from East and Southeast Asia (specifically Cambodia, Myanmar, Japan and southern China), analyzed whole genome data from individual *S. stercoralis* worms in addition to the *SSU* and *cox*1 markers [16, 20, 23]. Based on the mitochondrial *cox*1 sequences, *S. stercoralis* in Southeast Asia appears to be very genetically diverse, belonging to a large geographically widespread population, as is illustrated by the fact that many worms isolated from Cambodia [16], Laos [17], Myanmar [18] or Thailand [19], appear more closely related to some individuals isolated on a different continent than to some other worms isolated from the same country or even the same village. Even if only worms isolated from humans are considered, *S. stercoralis* shows a considerable diversity in the *SSU* HVR I and IV, loci that in other nematodes appear to be fairly uniform within a given species [16, 18, 20, 24]. Interestingly, except for a presumably asexual population in southern China that might have arisen by a rather recent hybridization event [20], very few [18] or no [16, 24] individuals heterozygous for different *SSU* haplotypes were found. Nevertheless, whole genome data did not support the hypothesis that different *SSU* haplotypes represent reproductively isolated populations [16].

In Thailand, *S. stercoralis* is prevalent in several regions of the country and it is recognized as a serious medical problem. Recently, the prevalence of *S. stercoralis* infections in the northern, central, northeastern and southern regions were estimated at 0.9–15.9% [25, 26], 2.47% [27], 22.80–32.80% [28, 29] and 0.9–1.7% [30, 31], respectively. To our knowledge, there are two studies providing molecular information about human derived *Strongyloides* spp. from northeastern Thailand [19, 32]. One of these [19] studied people exposed to non-human primates in two provinces (Maha Sarakham and Udon Thani), and one worm from each positive patient was analyzed. Based on the *SSU* HVR IV the authors identified as *S. fuelleborni fuelleborni* one out of the 19 *Strongyloides* spp. worms isolated. Although this parasite is prevalent in long-tailed macaques in this country [33], this was the first report of a human *S. fuelleborni fuelleborni* infection in Thailand. The other 18 worms were identified as *S. stercoralis.* While all of them had the same *SSU* haplotype, namely HVR IV haplotype A (according to the nomenclature in [16, 22]), at the *cox*1 locus, the samples fell into 13 different haplotypes. More recently, the *cox*1 gene and the *SSU* HVR IV of individual *Strongyloides* isolated from humans and dogs in two villages of the Kalasin province were sequenced [32]. The authors found that each of the 28 adult worms sequenced from humans (*n* = 23) and dogs (*n* = 5) possessed the *SSU* HVR IV haplotype A. At *cox*1, the sequences represented eight different haplotypes, of which four were new haplotypes, and found that dogs and humans shared the same haplotypes.

In this study we analyzed the *SSU* HVR I and IV, the *cox*1 and the whole genome sequences of *Strongyloides* spp. isolated from northern and western Thailand. All the *Strongyloides* specimens tested were *S. stercoralis*, confirming that human infections with *S. fuelleborni fuelleborni* are rare. All worms were phylogenetically very close to other samples from the Southeast Asian peninsula and provided no evidence for genetic sub-structuring within Thailand or on the Southeast Asian peninsula. Similar to other samples from this region, these new samples from Thailand showed low heterozygosity, when compared with samples recently described from Japan and southern China [20, 23], further supporting the hypothesis that *S. stercoralis* on the Southeast Asian peninsula form one intermixing population.

## Methods

### Collection of *S. stercoralis*

Fresh fecal samples were collected from residents living in the Chiang Mai and Lamphun provinces in northern Thailand and were examined for *S*. *stercoralis* infections by using modified kato-katz technique [34], and the modified Baermann technique [21, 35]. In addition, 12 *S*. *stercoralis* positive samples of patients living in four provinces in northern Thailand (Chiang Mai, Lamphun, Lampang and Mae Hong Son) and one province (Tak) in western Thailand were included in this study. All patients had visited the Maharaj Nakhon Chiang Mai Hospital between June and November 2018. Fecal samples were examined by the direct fecal smear technique [35] at the laboratory of the Department of Parasitology, Faculty of Medicine, Chiang Mai University. To isolate the worms, the samples were cultured by the Harada-Mori technique [35] at ambient temperature for 48–72 h; 95% ethanol was added to the cultured samples and they were centrifuged at 1500 *rpm* for 5 min. After removing the ethanol, worm pellets were transferred and preserved in 95% ethanol at − 20 °C until molecular analysis. The worms were preserved in ethanol as batches (one batch per patient), and later picked individually into 10 μl of water and frozen, as described by Zhou et al. [21].

### Worm lysis, *SSU* and *cox*1 genotyping

Genotyping at the *SSU* HVR I, HVR IV and the *cox*1 loci were performed as described by Zhou et al. [21]. In brief, worms [first-stage larvae (L1s) for field samples and infective third-stage larvae (iL3s) for clinical samples] stored in 10 μl water were frozen and thawed 3 times. Ten microliters of 2× lysis buffer (20 mM Tris-HCl pH 8.3, 100 mM KCl, 5 mM MgCl_2_, 0.9% NP-40, 0.9% Tween 20, 240 μg/ml Proteinase K) were added and the samples incubated at 65 °C for 2 h. The worm lysate was then either used directly for genotyping or stored at − 20 °C. Using 2.5 μl of worm lysate as a template, PCR amplification of *SSU* HVR-I, *SSU* HVR-IV and *cox*1 with Taq DNA polymerase (Cat. # M0267; New England BioLabs, Ipswich, USA) was carried out according to the manufacturer’s protocol. The PCR cycling program was as follows: an initial denaturation step at 95 °C for 60 s; followed by 40 cycles of denaturation at 95 °C for 20 s, annealing for 15 s and extension at 68° C for 90 s; followed by a final extension step at 68 °C for 5 min. The primers and the respective annealing temperatures are listed in Table 1.

**Table 1:** Primers, annealing temperatures and product size

Region amplified	Primer		Sequence (5′–3′)	Annealing T (°C)	Product size (bp)
*SSU* HVR-I	Forward Reverse Sequencing	ZS6492 RS5402 ZS6492	AAACATGAAACCGCGGAAAG CATTCTTGGCAAATGCTTTCG AAACATGAAACCGCGGAAAG	50	825
*SSU* HVR-IV	Forward	18SP4F	GCGAAAGCATTTGCCAA	57	712
	Reverse	18SPCR	ACGGCCGGTGTGTAC		
	Sequencing	ZS6269	GTGGTGCATGGCCGTTC		
*cox*1	Forward	ZS6985	GGTGGTTTTGGTAATTGAATG	47	837
	Reverse	ZS6986	ACCAGTYAAACCACCAATAGTAA		
	Sequencing	ZS6990	GGTTGATAAACTATAACAGTACC		

*Note*: For more information and references see [21]

*Abbreviation*: T, temperature

### Sequence analysis

The *SSU* and *cox*1 sequences were analyzed with SeqMan Pro version 12 (Lasergene package; DNAStar, Inc., Madison, WI, USA) and checked manually. For *S*. *stercoralis cox*1, the same 552-bp product as described in [16] was considered. The *cox*1 sequences were aligned and phylogenetic analysis was performed as described previously [16] with MEGA7 [36] using ClaustalW for alignment and the neighbor-joining (NJ) and the maximum-likelihood (ML) methods for tree reconstruction, with default settings. The sequence of *Necator americanus* (GenBank: AJ417719) was used as the outgroup. For comparison, selected published *cox*1 sequences were also included in the analysis. The corresponding accession numbers and references are listed in the respective figure.

### Whole genome sequencing of individual *S. stercoralis*

Genomic libraries of 15 *S. stercoralis* (5 first stage larvae, 10 infective larvae) were prepared following the method described previously [21]. In brief, genomic DNA was cleaned up by mixing 20 μl worm lysate with 4 μl magnetic SpeedBeads^TM^ (Sigma-Aldrich, St. Louis, USA) and 16 μl bead buffer (18% PEG8000, 2.5 M NaCl, 10 mM Tris-HCl pH 8.0, 1 mM EDTA pH 8.0). After DNA clean-up, the DNA was tagmented by mixing 7 μl DNA with 2 μl H_2_O, 2 μl 5× TAPS-DMF buffer (50 mM TAPS, 25 mM MgCl_2_, 50% DMF), and 1 μl Tn5 (purchased from Illumina (San Diego, USA) and diluted 1:100 in dialysis buffer -100 mM HEPES-KOH pH 7.2, 0.2 M NaCl, 0.2 mM EDTA, 0.2% Triton X-100, 20% glycerol). PCR amplification, adapter extension and barcoding were performed by adding 5 μl 5× Q5 reaction buffer, 1 μl 10 mM each dNTPs, 1 μl each of 5 μM Nextera i5 and i7 primer, 0.25 μl Q5 high-fidelity DNA polymerase (Cat. # M0491S; New England BioLabs, Ipswich, USA) and 19.75 μl H_2_O to the mixture, followed by the thermocycling program: 72 °C for 4 min, 98 °C for 30 s; followed by 17–18 cycles of denaturation at 98 °C for 15 s, annealing at 67 °C for 20 s, extension at 72 °C for 90 s and cooling to 4 °C. The 300–600 bp fragments of PCR products were selected with beads. Libraries were quantified on an Agilent 2100 Bioanalyzer and then sequenced on a Illumina HiSeq 3000 instrument (150-bp paired-end) at the Genome Core facility at the MPI for Developmental Biology (Tübingen, Germany). As part of their service, sequencing adapter sequences were removed by the Genome Core facility staff using the bcl2fastq software (version 2.18.0.12) with user defined parameter barcode-mismatches set to 1. No additional quality filtering was applied. Potential duplicate reads resulting from PCR amplification were removed by the software samtools-rmdup.

### Analysis of the whole genome data

#### Alignment

Raw reads were mapped to the *S. stercoralis* reference genome (version PRJEB528.WBPS11 from WormBase ParaSite) using bwa mem with default settings [37]. Additional file 1: Table S1 shows an overview of sequencing and alignment steps. In addition to the 15 individual *S. stercoralis* sequenced in this study, we also included the published whole genome sequences of selected wild isolates from China [20], Cambodia [16], Myanmar and Japan [18, 23] for comparison. To date, these are the only 4 countries from where the whole genomic sequences of individual wild-collected *S. stercoralis* have been published. Details of sample selection: for Cambodia, Myanmar and Japan, we have selected the same samples (3 and 4, respectively) as included in [20]; for China, we selected only 1 sample, namely the one with the lowest genomic heterozygosity. Since only the homozygous variants were used for the phylogenetic analysis, including more samples with high heterozygosity would have resulted in a large decrease of the number of informative sites.

#### Variants calling

Variants deviating from the *S. stercoralis* reference genome were called as described previously [38]. In brief, raw variants were called using the mpileup, bcftools, and vcfutils.pl programs of the samtools suite (version 0.1.18) [39] and filtered for variants with quality values ≥ 20. These variant calls integrate information such as base quality, coverage, and alignment quality. The accuracy of this procedure was previously assessed by comparison with whole genome Sanger sequencing data yielding an agreement of 98% [38]. For analysis of population structure, all variant sites were pooled and called in all samples in order to get the full genotypic data including reference alleles.

#### Population structure

The genome-wide phylogeny was computed by the neighbor-joining method as implemented in the *phangorn* package in R [40] and is based on 1315 variant sites (sites with indels in any of the samples were ignored) that were called as homozygous in all samples (see [38, 41] for further details).

#### Heterozygosity

To estimate heterozygosity, we defined heterozygous sites based on a positive FQ value and a quality value ≥ 20 in the vcf file that was generated by samtools, we then divided the number of heterozygous sites by the total amount of autosomal and sex chromosomal contigs in the *S. stercoralis* assembly. Only samples comprising > 80% of genomic regions with at least 15× depth were included.

## Results and discussion

### Phylogenetic position of the new samples based on the *SSU* and *cox*1

First, we determined the *SSU* HVR I, HVR IV and *cox*1 sequences of the newly isolated samples from northern and western Thailand and compared them with each other and with previously published sequences. All worms had *SSU* HVR IV haplotype A (nomenclature as proposed by [16, 22]), indicating that they all were *S. stercoralis* and not *S. fuelleborni*. Among the 144 worms representing 16 different persons (1–15 worms per host individual), we found two different HVR I haplotypes (Table 2), namely haplotype I, which was proposed to be the ancestral haplotype [16] and haplotype II, which was the most common haplotype among *S. stercoralis* from humans in northern Cambodia [16, 24]. Similarly, haplotype II was the predominant haplotype (138 of 144 worms) in this study. Haplotype I was found in only 2 of the 16 patients, and within these patients, in 6 out of 9 worms.

**Table 2:** *Cox*1, *SSU* HVR-I and HVR-IV haplotypes of *S. stercoralis* from northern and western Thailand

Sample ID	Source	Location (District/Province)	*cox*1 haplotype^c^	*SSU* HVR-I + HVR-IV haplotype^d^	
				I + A	II + A
S9	Residents^a^	Doi Lo/Chiang Mai	H6 (1), H8 (15)	–	15
S10		Doi Lo/Chiang Mai	H9 (16)	–	15
S12		Mae Tha/Lamphun	H9 (12), H11 (2), H12 (1)	2	6
S13		Mae Tha/Lamphun	H3 (10), H10 (5)	–	11
S14	Hospital patients^b^	San Pa Tong/Chiang Mai	H1 (21)	–	9
S16		Mae On/Chiang Mai	H10 (13)	–	6
S17		San Pa Tong/Chiang Mai	H1 (15)	–	9
S19		Mueang/Chiang Mai	H9 (16)	4	3
S20		Pai/Mae Hong Son	H7 (16)	–	8
S21		Chiang Dao/Chiang Mai	H2 (16)	–	9
S22		Mae Sot/Tak	H5 (13)	–	1
S23		Mae On/Chiang Mai	H10 (14)	–	13
S24		Mueang Pan/Lampang	H4 (3), H12 (12)	–	9
S25		na	H10 (15), H12 (1)	–	8
S27		Mae Tha/Lamphun	H10 (1), H12 (12)	–	7
S28		Doi Lo/Chiang Mai	H12 (15)	–	7

^a^Clinically healthy people sampled at home

^b^Hospital patients parasitologically surveyed as part of the routine procedures of the hospital. These patients were not admitted to the hospital because of strongyloidiasis, but for other reasons

^c^HX indicates the haplotype number, the number in parenthesis indicates in how many individuals the particular haplotype was found. For the sequences of the haplotypes see GenBank accession numbers MN871994-MN872230

^d^For the nomenclature see [16, 22]. Note that the numbers of worms for which the *cox*1 and the *SSU* sequences were successfully determined are not identical

**Fig. 1:**
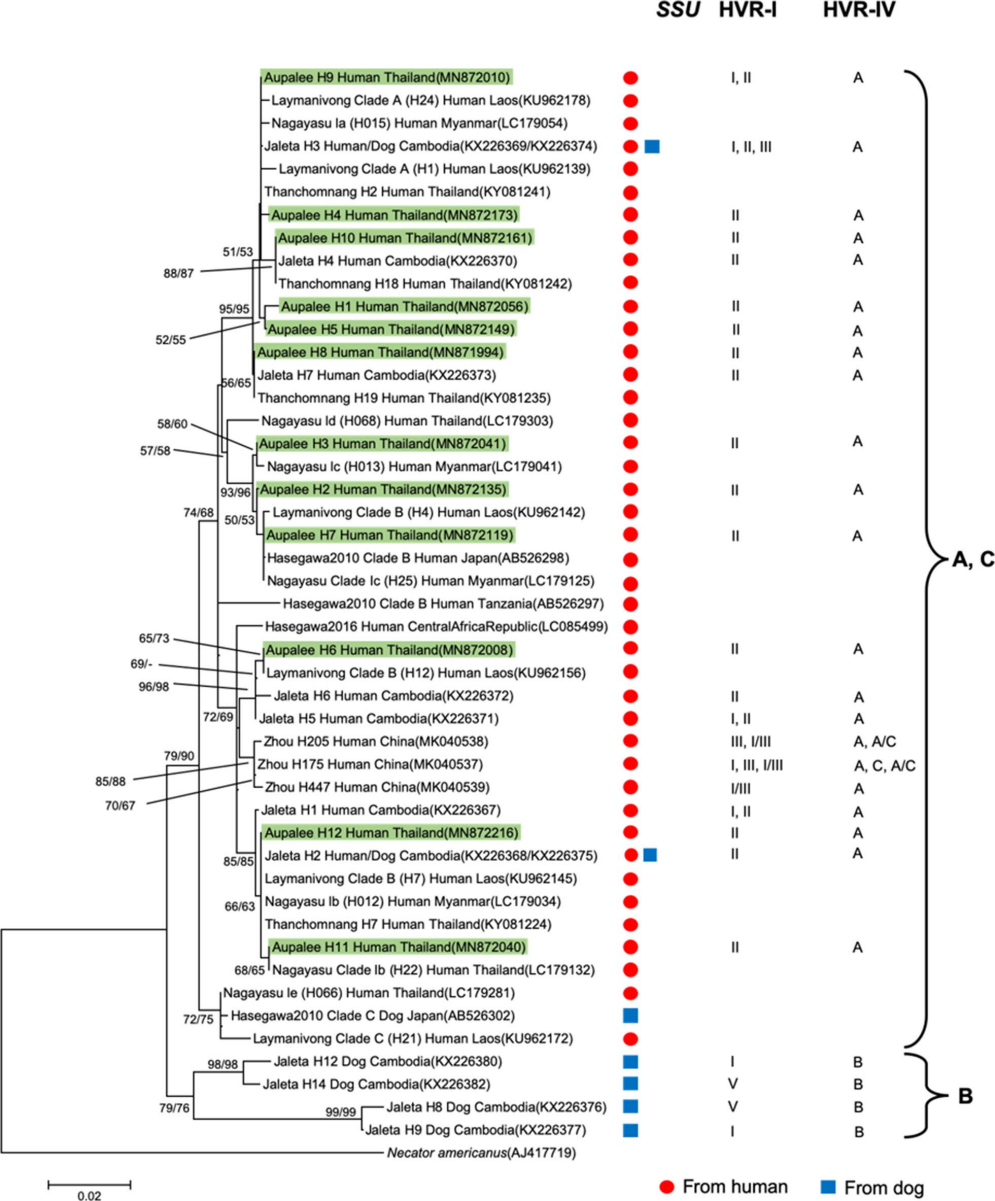
*cox*1 gene neighbor-joining tree of different *S. stercoralis* isolates based on 552-bp partial sequences. Shown are the sequences found in this study (green box) and selected published *S. stercoralis* haplotypes representing the major phylogenetic groups described in recent *S. stercoralis cox*1 phylogenies. The scale-bar represents 0.02 substitutions per site. The bootstrap values represent 1000 bootstrapping repetitions. Bootstrap values for neighbor-joining and maximum likelihood analysis are shown above or near the branches. The labels are composed as follows: author of the reference; haplotypes names according to this reference; host the isolate was derived from; country the isolate was isolated from; GenBank accession number. The host a particular sequence was found in is further highlighted with a red filled circle for “human” and a blue filled square for “dog”. The two columns on the right indicate the *SSU* HVR-I and HVR-IV haplotypes found among the worm individuals of the respective *cox*1 haplotype according to the respective authors, if known. Published sequences can be found under the following references [14–20]

Partial sequences (552 bp) of the mitochondrial *cox*1 gene were obtained from 237 *S. stercoralis*, representing all four positive human hosts in the village and twelve of the patients from a local hospital. In total, 12 haplotypes were identified in this study. Five of these haplotypes (H1-H5) had not been reported before in *S. stercoralis*, three haplotypes (H6, H7, H11) have been observed before but not in Thailand and the remaining four haplotypes have been observed in *S. stercoralis* in Thailand (Table 2). Interestingly, all six worms with *SSU* HVR haplotype I shared the same *cox*1 haplotype (H9). To examine the phylogenetic relationships, we reconstructed neighbor-joining and maximum-likelihood trees with our data and selected published *cox*1 sequences [20] (Fig. 1). Our *cox*1 haplotypes distributed among the ones found in Southeast Asian countries (Thailand, Laos, Myanmar and Cambodia) and did not group into different clades according to their country of origin.

### Whole genome analysis

**Fig. 2:**
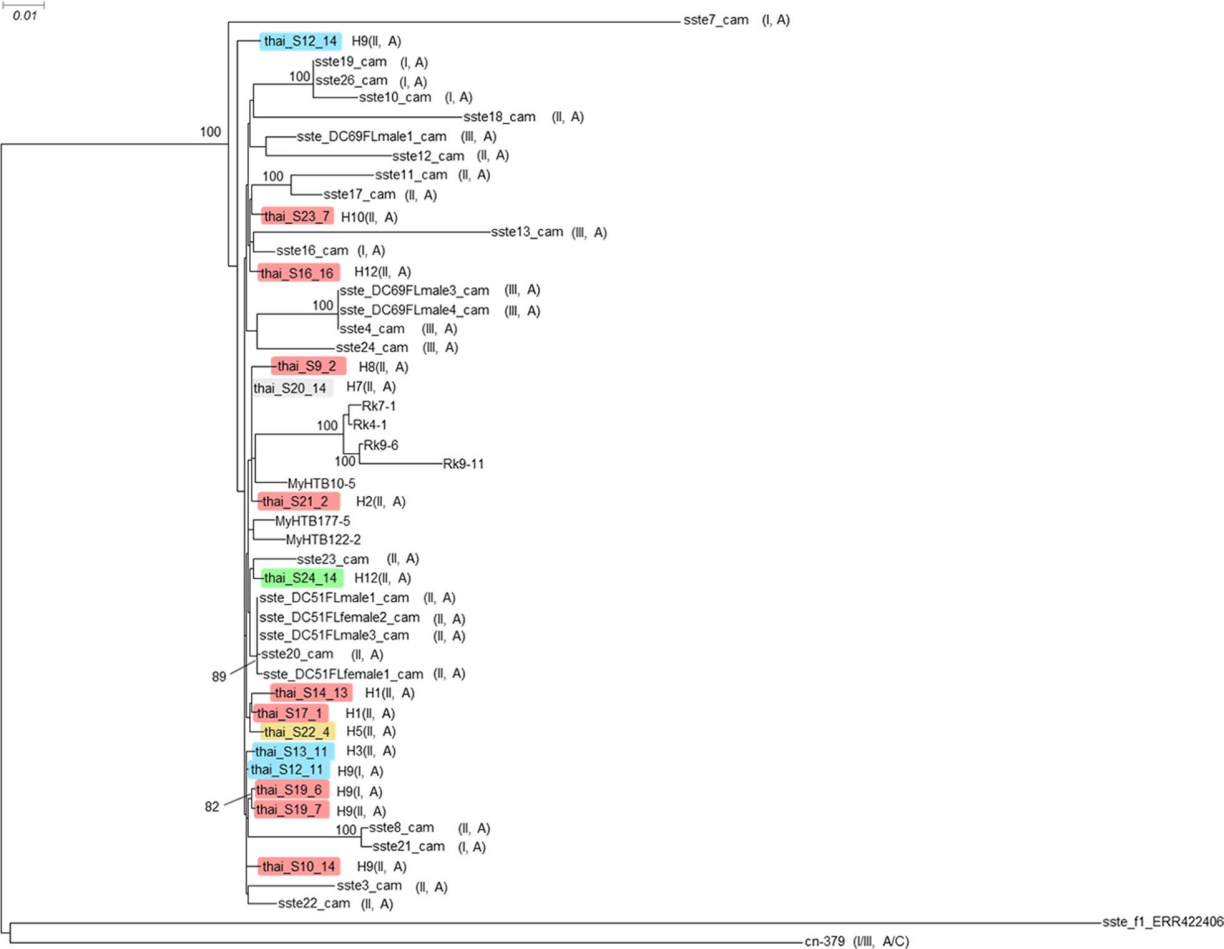
Phylogenetic tree based on whole genome sequences. Sequences newly determined for this study are highlighted in color, followed by the *cox*1 haplotype number and the *SSU* HVR-I and HVR-IV haplotypes in parentheses. The colors indicate the provinces: red, Chiang Mai; blue, Lamphun; green, Lampang; grey, Mae Hong Son; yellow, Tak. For comparison, selected published *S. stercoralis* whole genome sequences described in recent whole genome-based *S. stercoralis* phylogenies are shown (not highlighted). Samples ending with “cam” are from Cambodia [16]. Samples starting with “My” and “Rk” are from Myanmar and Japan, respectively [23]. Sample cn-379 is from southern China [20]. sste_f1_ERR422406 is a free-living female of the *S. stercoralis* reference isolate [42]. If listed in the corresponding reference, the *SSU* HVR-I and HVR-IV haplotypes are indicated in parentheses (note that HVR-IV haplotype C in [20] is the same as HVR-IV haplotype E in [12]; this was noticed by Barratt et al. [22] and occurred because the two publications appeared almost simultaneously). Key for comparing samples from Cambodia with figure 4 of [20]: sste3_cam to sste26_cam correspond to Kh-3 to Kh-26; sste_DC51FLfemale1_cam = Kh-27; sste_DC51FLfemale2_cam = Kh-28; sste_DC51FLmale1_cam = Kh-29; sste_DC51FLmale3_cam = Kh-30; sste_DC69FLmale1_cam = Kh-31; sste_DC69FLmale3_cam = Kh-32; sste_DC69FLmale4_cam = Kh-33

**Fig. 3:**
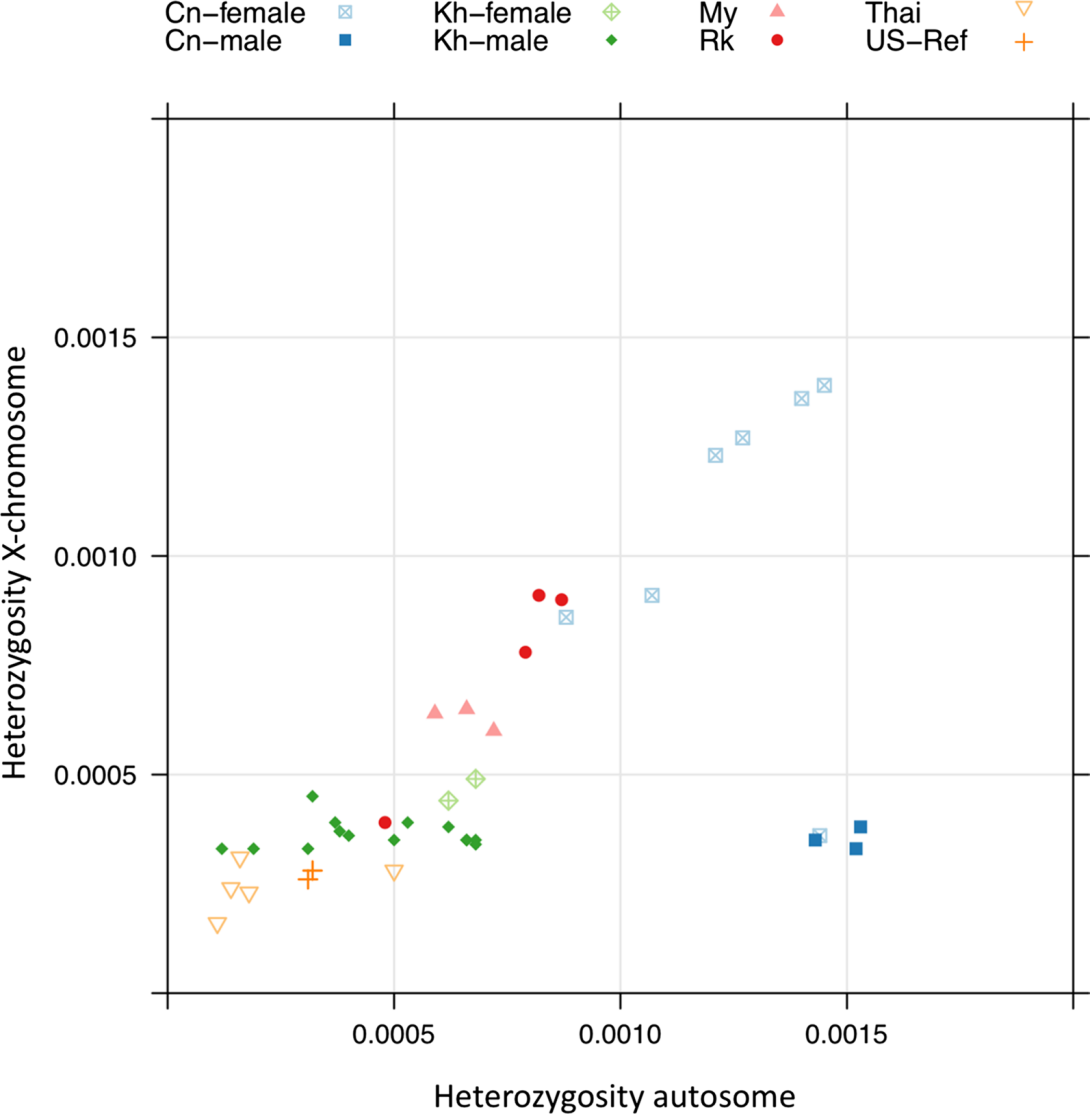
Heterozygosity of individual *S. stercoralis*. The heterozygosity on the autosomes is plotted against the heterozygosity on the X chromosome. For comparison, published data from *S. stercoralis* individuals from different geographical locations are included. Cn, China [20]; Kh, Cambodia [16]; My, Myanmar; Rk, Japan (both [23]); Thai, Thailand (present study); US-Ref, USA-derived reference laboratory strain PV001 [42]. For Cn, Kh, My, Rk and the USA reference, the same samples as in Fig. 5 of [20] are included. For Thailand, all samples in Fig. 2 that fulfilled the read coverage criteria (see Methods) were included in this analysis

For 15 worms from northern Thailand, we determined the genome sequences and compared them to the published data (Fig. 2). First, we conducted a phylogenetic analysis based on the whole genome sequences. In full agreement with the mitochondrial *cox*1 data, the worms from Thailand grouped clearly with the other worms from the Southeast Asian peninsula without forming their own subgroup. In two cases, we sequenced worms from the same patient but found a different *SSU* HVR I haplotype (haplotypes I and II). In one of these cases (patient S19), the genomes of the two worms were almost identical, suggesting that these two worms must have been very closely related. However, in the other case (patient S12), the two worms were comparably far apart, with the worm that had the common *SSU* HVR I haplotype II (S12_14) being removed from all the others, while the other with the comparatively rare *SSU* HVR I haplotype I (S12_11), grouping very closely with other samples. This result represents additional very strong evidence that the different *SSU* HVR I haplotypes do not represent reproductively isolated populations [16, 20]. As an additional genomic feature, we studied the heterozygosity of the individual worms, because previous studies had found differences in heterozygosity between samples from the Southeast Asian peninsula and populations in Japan [23] and southern China [20], which presumably reproduce largely or exclusively asexually (Fig. 3). We found that the new samples from Thailand clustered with the other samples from the Southeast Asian peninsula, generally towards the lower end of this group, particularly in respect to the heterozygosity on the X-chromosome.

## Conclusions

Similar to an earlier study in Cambodia [16], we found that different *SSU* HVR I haplotypes were not indicative for genetic isolation as judged by whole genome sequencing. This leaves open the question, as to why, as in Cambodia, we did not find individuals heterozygous for different *SSU* HVR I haplotypes. Based on nuclear *SSU*, mitochondrial *cox*1 and whole genome sequence information, *S. stercoralis* in northern and western Thailand appear to be part of a genetically diverse continental Southeast Asian population. This is in contrast to two presumably genetically more isolated local populations recently described in Japan [18, 23] and southern China [20] and rather suggests strong mixing of *S. stercoralis* over the entire mainland Southeast Asian region.

## Supplementary information

**Additional file 1: Table S1.** Overview of sequencing data. The first two columns denote sample names and accessions in the European Nucleotide Archive. The third column indicates the total number of raw reads (paired end reads are counted as two reads). The fourth column indicates the number of aligned reads. The numbers of aligned may vary substantially due to different levels of contamination by bacteria and host tissue.

## Data Availability

The *cox*1 DNA sequences obtained in this study are available under the GenBank accession numbers MN871994-MN872230. The whole genome sequencing data are available under the GenBank SRA accession number PRJNA602131. All other relevant data is within the manuscript and its additional file.

## Abbreviations

HVR: hypervariable region (within the *SSU*)
iL3: infective third stage larva
NTD: neglected tropical disease
*SSU*: small ribosomal subunit rDNA (encodes the *18S* ribosomal RNA
STH: soil-transmitted helminthiasis
