# Supplementary material for: Genomic studies on Strongyloides stercoralis in northern and western Thailand
Source: Parasit Vectors. 2020 May 13;13:250. doi: 10.1186/s13071-020-04115-0 (PMC7222524; doi:10.1186/s13071-020-04115-0)
Supplement: Supplementary file 1 — Additional file 1: Table S1. Overview of sequencing data. The first two columns denote sample names and accessions in the European Nucleotide Archive. The third column indicates the total number of raw reads (paired end reads are counted as two reads). The fourth column indicates the number of aligned reads. The numbers of aligned may vary substantially due to different levels of contamination by bacteria and host tissue. [file 13071_2020_4115_MOESM1_ESM.docx]

**Additional file 1: Table S1.** Overview of the sequencing data

| **Sample** | **European Nucleotide Archive (Sample ID)** | **Number of reads** | **Aligned reads** |
| --- | --- | --- | --- |
| thai_S10_14 | SAMN13881925 | 37803064 | 4667213 |
| thai_S12_11 | SAMN13881926 | 27800408 | 12134688 |
| thai_S12_14 | SAMN13881927 | 30934640 | 7398826 |
| thai_S13_11 | SAMN13881928 | 32009804 | 6312215 |
| thai_S14_13 | SAMN13881929 | 26073764 | 22215146 |
| thai_S16_16 | SAMN13881930 | 30557288 | 25960641 |
| thai_S17_1 | SAMN13881931 | 28275474 | 25315408 |
| thai_S19_6 | SAMN13881932 | 24017238 | 21315548 |
| thai_S19_7 | SAMN13881934 | 18115622 | 10654943 |
| thai_S20_14 | SAMN13881933 | 27197176 | 22252749 |
| thai_S21_2 | SAMN13881935 | 17615384 | 16336521 |
| thai_S22_4 | SAMN13881936 | 32499184 | 27280552 |
| thai_S23_7 | SAMN13881937 | 26199044 | 23946859 |
| thai_S24_14 | SAMN13881939 | 31358008 | 27170274 |
| thai_S9_2 | SAMN13881924 | 40570024 | 31805658 |
